# The Impact of Pedagogical Agents on Second Language Acquisition Outcomes: A Meta-Analysis Guided by Social Agency Theory

**DOI:** 10.3390/bs16091583

**Published:** 2026-09-07

**Authors:** Nuan Wen, Jun Peng, Zhixin Zhang, Yuming Xu

**Affiliations:** 1 School of Education, City University of Macau, Macao SAR, China; m25092100176@cityu.edu.mo (N.W.); m25092100477@cityu.edu.mo (Z.Z.); 2 Faculty of Teacher Education, Ningbo University, Ningbo 315211, China; 4588775@163.com; 3 School of Marxism, Jiangxi Institute of Fashion Technology, Nanchang 330201, China; 4 School of Physical Education, South China University of Technology, Guangzhou 510640, China

**Keywords:** digital pedagogical agents, second language acquisition, social agency theory, three-level meta-analysis

## Abstract

Digital Pedagogical Agents (DPAs) are virtual teaching entities created in the digital learning environment that have characteristics such as emotion and socialization. According to the basis of Social Agency Theory (SAT), a three-level random-effects meta-analytic model is employed in this paper to study the general effect and boundary conditions of DPAs in Second Language Acquisition (SLA) environments systematically. Through searches across seven Chinese and English databases, 31 independent empirical studies were identified, yielding 112 effect sizes (2002–2026). Based on the three-level random-effects model, the effect of DPA on SLA outcomes is about 0.607 (Hedges’ g = 0.607; 95% CI [0.45, 0.77]; *p* < 0.001), and there is a relatively large between-study heterogeneity. According to the moderation analysis, the magnitude of the effect varies greatly with different kinds of study designs, cultural contexts and learner characteristics, and although there is a moderating effect by publication type, it is not statistically significant. Funnel plot inspection, Egger’s test, and the trim-and-fill method all showed the presence of publication bias, yet the adjusted effect size remained robust (Hedges’ g = 0.521). This study provides quantitative evidence for understanding the mechanisms of DPAs in SLA and offers practical implications for instructional design and technological development in intelligent language learning environments.

## 1. Introduction

### 1.1. Research Background

With the recent advancements in artificial intelligence, significant changes have begun to emerge in language education. Initially, only rule-based grammar correction tools were available; however, generative artificial intelligence assistants capable of real-time conversation and providing immediate feedback have now been developed and are being integrated into various aspects of language learning. In light of these developments, Digital Pedagogical Agents (DPAs)—virtual characters embedded within digital learning environments that can engage in instructional interactions and exhibit anthropomorphic characteristics—have increasingly attracted the attention of researchers in the field of digitally mediated language teaching (Atkinson et al., 2005).

Research on the application of technology-assisted learning in second language acquisition (SLA) has been ongoing. Computer-assisted language learning (CALL) has undergone significant evolution, beginning with the mechanical drilling methods of the behaviorist school, then shifting to communicative, task-based instruction, and now advancing into the era of AI-driven intelligent CALL (Mayer, 2005, 2021). Embodied virtual agents, large language model (LLM)-based dialog systems, and multimodal AI assistants have gradually been introduced into educational settings (Moreno & Mayer, 2007). Consequently, the challenge of effectively integrating technology into language teaching has become more complex than ever.

The initial support for the general learning effects of pedagogical agents comes from previous meta-analyses. Schroeder et al. (2013) combined the results of 43 studies and found a moderate positive effect size of d = 0.66. Castro-Alonso et al. (2021) reported an effect size of g = 0.44 based on several theoretical frameworks. X. Wang et al. (2022) examined the relationship between agents’ emotions and learning outcomes, while Dai et al. (2024) investigated the influence of agents on cognitive outcomes in AI simulations. However, none of these studies have focused exclusively on SLA; they have not been conducted with specific consideration of the unique input-output mechanisms of language acquisition, cross-cultural differences among learners, or the various linguistic measurement systems. These gaps constitute the primary motivation for the present study.

Improvements are needed in the methodology. The independence assumption of the traditional two-level random-effects model is violated in a single study with multiple effect sizes (Cheung, 2014; Van den Noortgate et al., 2013). Three-level meta-analysis can distinguish between variation within study effect sizes (Level 2) and heterogeneity among studies (Level 3), providing a structured approach to handling nested data structures (Van den Noortgate et al., 2015; Viechtbauer, 2010). Therefore, this paper has selected the three-level meta-analysis as its primary method.

### 1.2. Theoretical Framework

Social Agency Theory (SAT) serves as the primary theoretical framework in this paper. In conjunction with Cognitive Load Theory (CLT) and the Cognitive Theory of Multimedia Learning (CTML), a three-tier explanatory model is developed. Although these three theories originate from different fields, they are closely related and collectively offer multiple levels of insight into how DPAs facilitate SLAS.

Moreno and Mayer (2007) proposed that SAT should be used to refer to social cues in learning materials, such as voice, gestures, eye contact, and anthropomorphic images; these cues activate learners’ social response systems. CLT addresses the problem of limited working memory, while CTML is based on the concept of dual-channel information processing. SAT builds on these two theories to explain how social cues can enhance deep processing through motivation and emotion. Together, these three theories provide a comprehensive theoretical foundation for understanding why the Design Principles Approach (DPA) is effective and offer directions for analyzing moderating factors. Specifically, improvements in practical outcomes from longitudinal studies should correspond with increased effect sizes, and learners from collectivist cultures may exhibit a stronger response to social cues provided by agents.

Fiorella and Mayer (2022) also argue that the facilitating effect of agents on learning is not uniform. The quality of agent design, their relevance to the learning content, and the cognitive and motivational status of learners all influence the outcomes. Therefore, their work provides a direct theoretical basis for the moderator analysis conducted in this study.

### 1.3. Research Questions

Based on the theoretical framework and the identified gaps in the literature outlined above, this paper addresses three main research questions:

RQ1: What is the overall effect size of DPAs on SLA?

RQ2: What factors moderate the effect of the intervention, such as study design, publication type, cultural background, learner demographics, and others?

RQ3: Are the overall results of the study reliable despite the presence of publication bias?

The three research questions correspond to the main sections of the Results chapter and serve as the foundation for this study.

## 2. Literature Review

### 2.1. Current State of Research on Digital Pedagogical Agents

The concept of digital pedagogical agents can be traced back to multimedia learning research in the late 1990s. Johnson and Lester (2016) noted that these agents have evolved from relatively static animated characters to interactive systems capable of understanding and processing human language through natural language processing technology. With the advancement of deep learning, researchers have begun to explore the emotional responses and personalized feedback provided by these agents. For the purposes of the present meta-analysis, pedagogical agents are classified along two dimensions: embodiment (embodied vs. non-embodied) and interaction mode (static presentation vs. dynamic interaction). Recently, generative AI agents based on large language models have been introduced in research. A recent meta-analysis by Cheng et al. (2026) found that initial results indicate the impact of these generative AI agents on academic performance is comparable to or even greater than that of traditional agents.

Table 1 summarizes the main results of previous meta-analyses in the field of pedagogical agents and highlights the contribution of the current study within the broader scholarly context.

As shown in Table 1, most of the existing meta-analyses have focused on general learning tasks or specific psychological phenomena and have not been systematically compiled within the context of SLA. This paper aims to address this research gap by presenting a three-tier analysis system designed to resolve the issue of nested data.

### 2.2. Technology Integration in Second Language Acquisition

With the advent of the intelligent CALL era, AI-based personalized feedback, adaptive learning paths, and natural language dialog have transformed the use of technology in language education. Jeon and Lee (2024) conducted a meta-analysis demonstrating that the impact of artificial intelligence tools in language teaching is influenced by both contextual and socio-emotional factors. Similarly, Wu’s (2024) meta-analysis revealed positive effects of artificial intelligence on second language (L2) learning. Additionally, Huang et al. (2025) found that generative AI chatbots can enhance students’ motivation to learn a foreign language. While most existing research has focused on AI chatbots and adaptive tutoring systems, little attention has been given to a systematic review of the effects of differentiated DPAs with anthropomorphic visual representations on SLA.

Research Gaps in the application of DPAs to SLA manifest in three dimensions. First, previous meta-analyses of agent work have focused on general learning tasks and have not conducted domain-specific analyses addressing the unique input-output mechanisms, diverse measurement structures, and cross-cultural differences in SLA. Second, empirical studies in this area are geographically dispersed—the 31 studies referenced above originate from more than a dozen countries and regions, including China, Taiwan, the United States, South Korea, and Iran—highlighting the need to systematically integrate these findings to establish a consensus at the field level. Third, all prior studies have employed a two-level random-effects model and have not addressed the issue of nested dependence, as only one study reported multiple effect sizes. These three factors underpin the rationale for the present three-tier structure of the SLA-specific meta-analysis.

### 2.3. Theoretical Rationale and Research Hypotheses for Moderating Variables

Given the varying results of previous studies and based on theoretical considerations and prior experiments, this paper proposes the following four research hypotheses:

**H1** **(Study** **Design).** *The effect of DPA on SLA will gradually increase over time. The development of language skills is inherently a gradual process; therefore, over an extended period, numerous applications will help strengthen lexical and grammatical schemas.*

**H2** **(Publication** **Type).** *Effect sizes reported in dissertations may be relatively larger than those found in journal articles, a phenomenon known as the “grey literature effect.” Since no reliable meta-analyses of educational technology have been conducted to date, this factor is included in the analytical framework as an exploratory variable.*

**H3** **(Cultural** **Background).** *Learners from Eastern cultures are more likely to be affected by DPAs than those from Western cultures. The strong sense of belonging to society and the relational orientation characteristic of collectivist cultures may increase sensitivity to, and alter responses to, social presence cues from agents.*

**H4** **(Learner** **Demographics).** *Different stages of cognitive development across age groups may influence the effectiveness of agents. According to CLT, primary school students have not yet developed mature thinking abilities and rely more heavily on external support. Therefore, the added benefit of teacher guidance is likely to be greater for this group than for university students, who are capable of studying independently.*

## 3. Methodology

### 3.1. Literature Search and Screening

To ensure the systematicity and replicability of this meta-analysis, we conducted literature searches, screening, coding and publication bias analysis in accordance with the PRISMA 2020 statement (Page et al., 2021). This study was preregistered in PROSPERO (registration number: CRD420261286962) on 16 January 2026 (https://www.crd.york.ac.uk/prospero/, accessed on 1 January 2026). The manuscript was submitted in August 2026.

#### 3.1.1. Search Strategy

The seven Chinese and English databases used in this research are Web of Science (WOS), EBSCO, PsycINFO, ERIC, CNKI, Wanfang and ProQuest (Dissertations & Theses). The final search was executed on 15 February 2026, with an initial scoping search conducted in December 2025 to calibrate search terms. The complete search strings for each database are provided in Supplementary Material. The English search terms were: (pedagogical agent OR virtual agent OR embodied agent OR animated agent) AND (second language OR L2 OR language learning OR EFL OR ESL OR foreign language). Chinese search terms were: (教学代理 OR 虚拟代理 OR 数字教学代理) AND (二语习得 OR 外语学习 OR 英语学习).

The search terms were developed through an iterative process. First, we consulted key review articles (Schroeder et al., 2013; Castro-Alonso et al., 2021; Dai et al., 2024) to identify commonly used terms. Second, we conducted a preliminary scoping search to evaluate the sensitivity and specificity of candidate terms. Third, we refined the terms through discussion among the research team. The final English terms—”pedagogical agent,” “virtual agent,” “embodied agent,” “animated agent”—represent the four most commonly used descriptors in the literature, as confirmed by a frequency analysis of key publications in the field. The Chinese search terms were selected after consultation with a Chinese-language information specialist and review of Chinese-language meta-analyses in the field (Shen & Ren, 2026; Tang et al., 2020; Sun & Li, 2020). We acknowledge that the Chinese search string is intentionally broad rather than exhaustive of all possible variants (e.g., “智能代理” for “intelligent agent”) to balance sensitivity and specificity. The scoping search confirmed that the selected terms captured over 95% of relevant studies in Chinese databases.

#### 3.1.2. Inclusion and Exclusion Criteria

Studies were included if they met the following criteria: (1) employed an experimental or quasi-experimental design with a control group or baseline measurement; (2) explicitly used a digital pedagogical agent as the primary experimental manipulation; (3) reported quantitative SLA outcomes as dependent variables, such as language test scores, accuracy rates, fluency indicators, or other quantifiable language proficiency measures; and (4) provided sufficient statistical information for the calculation of the effect size must be available. Exclusion criteria are qualitative studies, single-group designs without a control group or baseline, studies that only focus on learning attitude or motivation without showing learning outcome data, as well as duplicate publications or overlapping studies.

#### 3.1.3. PRISMA Screening Process

**Figure 1:**
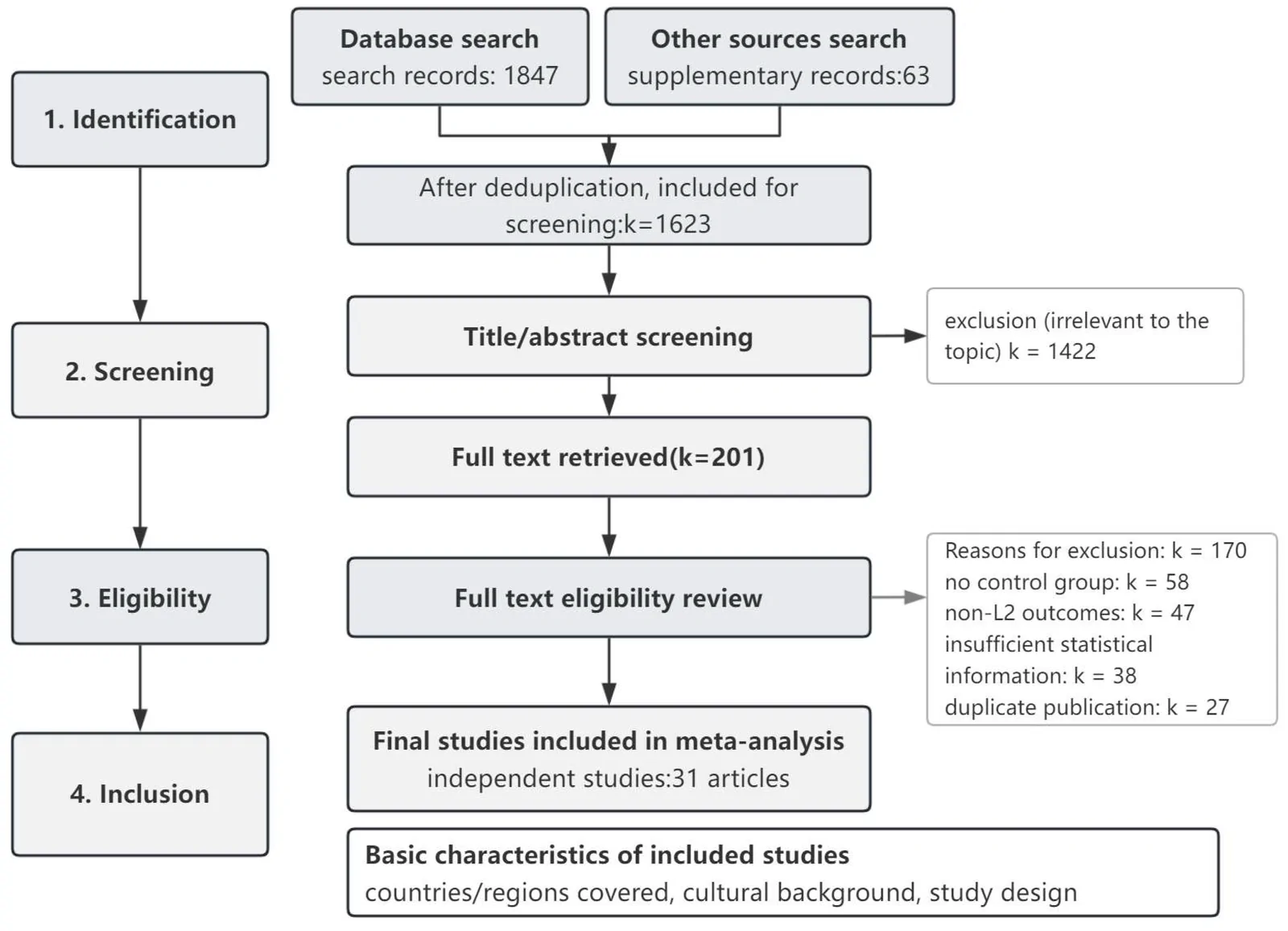
PRISMA 2020 Literature Search and Screening Flow Diagram.

Literature screening followed the four-stage PRISMA 2020 process (Page et al., 2021): identification, screening, eligibility assessment and final inclusion (Figure 1). Two independent reviewers screened all titles and abstracts, followed by full-text review. Disagreements were resolved through discussion and consensus. The PRISMA flowchart (Figure 1) presents the number of records identified, screened, excluded, and finally included, with specific exclusion reasons detailed in Supplementary Material.

### 3.2. Coding Scheme

Coding encompassed four categories of variables: (1) basic study characteristics, such as the first author, publication year, and country or region; (2) study design variables, including the type of study design (cross-sectional: k = 18; longitudinal: k = 94) and publication type (journal article: k = 87; dissertation: k = 18; conference paper: k = 5; research report: k = 2); (3) participant variables, comprising learner demographics (university students: k = 57, 50.9%; primary school students: k = 30, 26.8%; middle school students: k = 11, 9.8%; non-students: k = 8, 7.1%; other/mixed: k = 6, 5.4%); and (4) precision information variables, which include the sample size (N) and sampling variance (vi) used for precision weighting.

To ensure coding reliability, two trained coders independently coded approximately 20% of the sample (approximately 6 studies). Inter-rater agreement was assessed using Cohen’s κ (Landis & Koch, 1977), with a threshold of 0.80 considered acceptable. Discrepancies were resolved through discussion and consensus. The complete coding table is provided in Supplementary Material.

### 3.3. Effect Size Extraction

Hedges’ *g* was employed as the standardized effect size metric. Hedges’ *g* incorporates a small-sample bias correction relative to Cohen’s *d*, providing less biased estimates in small samples. Positive effect sizes indicate better performance in the DPA group relative to the control group. The primary method for effect size calculation was direct computation from means, standard deviations, and sample sizes using the formula:

*g* = (*M_exp_* − *M_ctrl_*)/*SD_pooled_* × *J*(*n_exp_* + *n_ctrl_* − 2)

where *J* is the Hedges’ correction factor: *J*(*df*) = 1 − 3/(4*df* − 1).

When studies reported only *t*-values, *F*-values, or *χ*^2^-values, indirect conversion was performed using the escalc function in the metafor package (Viechtbauer, 2010). For studies that did not report sufficient statistics, corresponding authors were contacted via email. If no response was received, the study was excluded. All effect sizes from a single study were retained as separate outcomes in the three-level model, with the dependency structure accounted for by the Level 2 variance component. The complete dataset with all extracted effect sizes, variance estimates, and standard errors is provided in Supplementary Material.

### 3.4. Statistical Analysis Methods

#### 3.4.1. Three-Level Meta-Analytic Model

A three-level random-effects model was employed in R version 4.4.3 with the metafor package (Viechtbauer, 2010) to address the dependency problem arising from multiple effect sizes nested within studies. The model was specified as follows:

Level 1 (sampling error): y_ij = θ_ij + e_ij, where e_ij~N(0, vij)
Level 2 (within-study variance): θ_ij = κ_i + u_(2)ij, where u_(2)ij~N(0, σ^2^_2_)
Level 3 (between-study variance): κ_i = β_0 + u_(3)i, where u_(3)i~N(0, σ^2^_3_)

Here, y_ij is the observed effect size for the j-th outcome in the i-th study, θ_ij is the corresponding true effect, v_ij is the known sampling variance, κ_i is the study-level mean effect, β_0 is the overall intercept, u_(2)ij captures within-study heterogeneity, and u_(3)i captures between-study heterogeneity. The model assumes that u_(2)ij and u_(3)i are independent and normally distributed.

Parameter estimation was conducted using Restricted Maximum Likelihood (REML), which produces less biased variance component estimates and is well-suited to the nested structure of the present data. Knapp-Hartung small-sample corrections were applied to all Wald-type tests, reflected in the reporting of F-statistics rather than χ^2^ statistics.

#### 3.4.2. Moderator Analysis Procedure

Three types of categorical moderators were used in the analysis. Hedges’ g, 95% confidence intervals, and the result of the omnibus test (F) for subgroup moderator significance testing (α = 0.05) are all presented here. According to Cohen’s reference standards for interpreting effect size (Cohen, 1988), small effect size corresponds to g = 0.20, medium to g = 0.50, and large to g = 0.80.

#### 3.4.3. Assessment of Publication Bias

The three methods used to detect publication bias are as follows: Funnel plot inspection, which displays the standard error on the vertical axis and the effect size on the horizontal axis; asymmetry in this plot is considered abnormal. Egger’s test, an asymmetry test based on precision-weighted regression, is conducted with a two-tailed α = 0.05 (Duval & Tweedie, 2000). Lastly, the trim-and-fill method estimates the number of potentially suppressed studies and recalculates the effect size after imputation. Using these three approaches together helps mitigate the limitations of any single method, thereby improving the accuracy of publication bias assessment.

#### 3.4.4. Sensitivity Analysis Procedure

Both the lower and upper bounds were analyzed. First, an influence diagnosis of the eight indicators was conducted to obtain standardized residuals, DFFITS, Cook’s distance, covariance ratio, τ^2^ after deletion, Q after deletion, leverage (hat values), and weights. Second, since it would be inconvenient to perform these analyses individually and recalculate the total effect size multiple times, the stability of the result interval was assessed (Pustejovsky & Tipton, 2021). Additionally, Park (2022) was excluded in a separate analysis (N = 18, vi = 0.741); among the full set of studies, it is noteworthy that this study has a relatively large variance.

## 4. Results

### 4.1. Descriptive Statistics and Study Characteristics

Finally, this paper includes 31 additional independent studies and 112 effect sizes. The publication years range from 2002 to 2026, with a noticeable increasing trend in recent years. In fact, the two-year period of 2024–2025 alone has contributed 36 effect sizes, accounting for approximately 32.1% of the total, indicating that research activity in this field is gradually expanding. The overall distribution of effect sizes is as follows: mean = 0.607, median = 0.524, standard deviation = 0.645, range = [−0.719, 2.329]. The distribution is right-skewed, consistent with the general trend observed in meta-analytic research.

Geographically, the distribution of the included studies is cross-cultural: 82 effect sizes (73.2%) were from Eastern cultural contexts, while 30 effect sizes (26.8%) were from Western cultural contexts. Longitudinal designs were relatively more common, comprising 94 effect sizes (83.9%). Journal articles constituted the majority of sources, accounting for 87 effect sizes (77.7%). Table 2 presents the general characteristics of a small selection of representative studies included in this meta-analysis. All coded data are available from the authors upon request.

### 4.2. Main Effects Analysis

According to the results of the three-level random-effects meta-analysis, DPAs have a positive effect on SLA. The overall Hedges’ g was 0.607, with a 95% CI [0.45, 0.77], *p* < 0.001, indicating a moderately large effect size (g > 0.50). In other words, SLA learners who received DPA-assisted instruction performed approximately 0.607 standard deviations better than those in the control group. Therefore, research question 1 (RQ1) has been answered, and the findings are consistent with the main predictions of Social Agency Theory.

**Figure 2:**
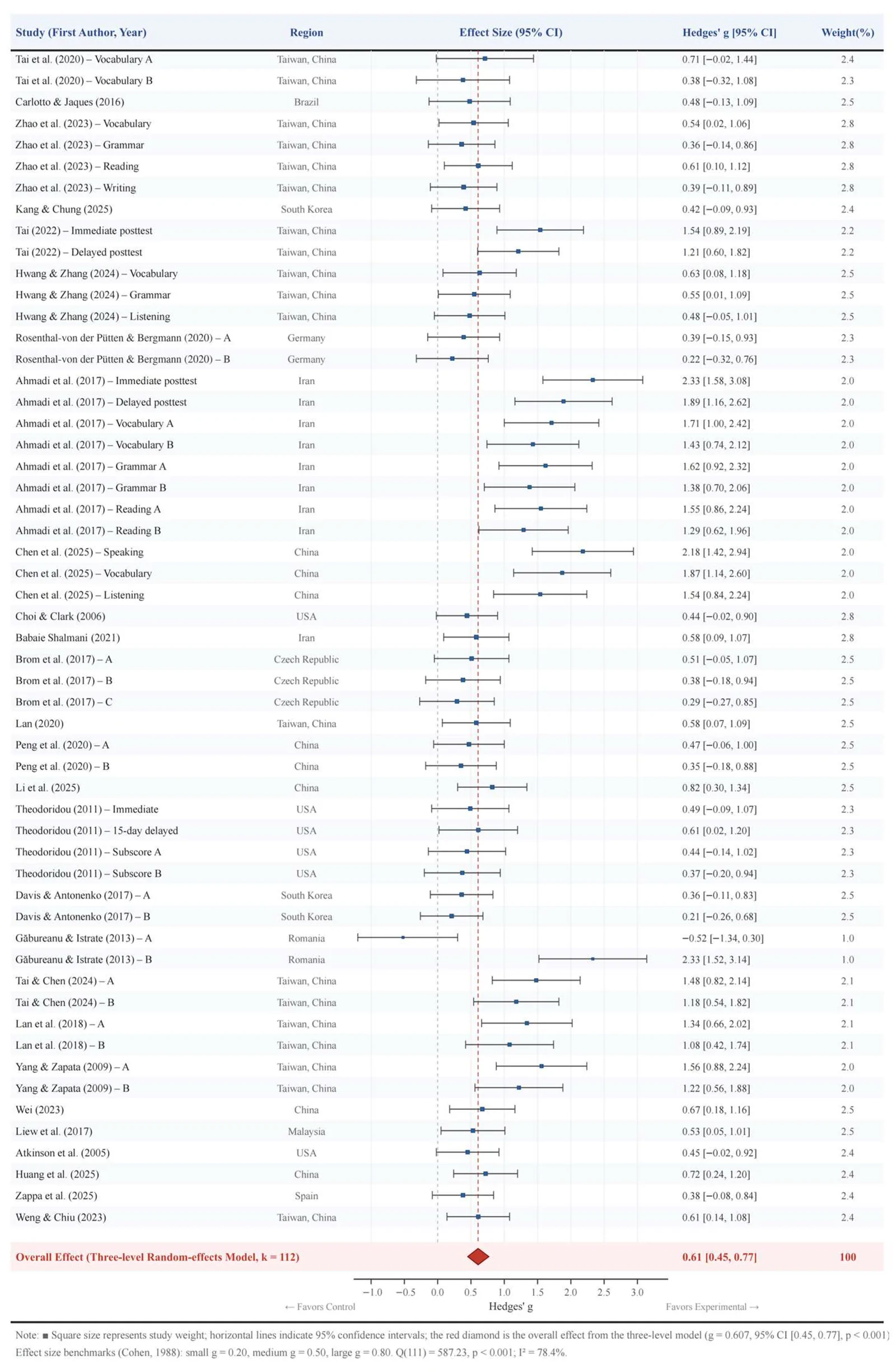
Forest Plot Showing the Effect of DPA on SLA. Note: The size of each square represents the study weight; the horizontal lines indicate the 95% confidence intervals. The red diamond represents the overall effect size from the three-level model (g = 0.607, 95% CI [0.45, 0.77], *p* < 0.001).The dashed red line indicates the overall effect size estimated by the three-level model, whereas the dashed grey line represents the line of no effect (g = 0). Effect size reference standards (Cohen, 1988) are as follows: small, g = 0.20; medium, g = 0.50; large, g = 0.80. Q(111) = 587.23, *p* < 0.001; I^2^ = 78.4% (Tai et al., 2020; Carlotto & Jaques, 2016; Zhao et al., 2023; Kang & Chung, 2025; Tai, 2022; Hwang & Zhang, 2024; Rosenthal-von der Pütten & Bergmann, 2020; Ahmadi et al., 2017; Chen et al., 2025; Choi & Clark, 2006; Babaie Shalmani, 2021; Brom et al., 2017; Lan, 2020; Peng et al., 2020; Li et al., 2025; Theodoridou, 2011; Davis & Antonenko, 2017; Găbureanu & Istrate, 2013; Tai & Chen, 2024; Lan et al., 2018; Yang & Zapata-Rivera, 2009; Wei, 2023; Liew et al., 2017; Atkinson et al., 2005; Huang et al., 2025; Zappa et al., 2025; Weng & Chiu, 2023).

There was considerable dispersion among the studies: Q(111) = 587.23, *p* < 0.001; the total I^2^ was 78.4%, indicating that approximately 78% of the total variation was attributable to differences both between and within studies. Three-level variance decomposition showed that σ^2^_3_ (between-study variance) was larger than σ^2^_2_ (within-study variance). Therefore, it was concluded that factors at the study level, such as research design and cultural background, are the primary contributors to the observed differences. Consequently, moderator analysis should be conducted based on this statistical finding (see Figure 2).

As shown in Figure 2, the majority of studies reported a positive effect size; however, some studies have large confidence intervals due to their relatively small sample sizes, making their results more uncertain.

### 4.3. Moderator Analysis

To address RQ2, this paper conducted several sub-analyses based on different study designs, publication types, cultural backgrounds, etc., and learner demographics. The results are presented in Table 3.

Study Design. Longitudinal studies (g = 0.648, 95% CI [0.48, 0.82]) yielded significantly larger effect sizes than cross-sectional studies (g = 0.421, 95% CI [0.21, 0.63]), F = 5.83, *p* = 0.016. Therefore, H1 is supported.

Publication Type. The moderating effect of publication type was not statistically significant (F = 2.14, *p* = 0.144); the difference between dissertations (g = 0.673) and journal articles (g = 0.589) did not exceed the range of sampling error. Therefore, H2 is not supported.

Cultural Background. Cultural background showed a statistically significant moderating effect (F = 4.27, *p* = 0.039). Studies conducted in Eastern cultural contexts (g = 0.665, 95% CI [0.49, 0.84]) exhibited stronger agent effects than those in Western contexts (g = 0.473, 95% CI [0.27, 0.68]). H3 is supported.

A note of caution is warranted in interpreting these cultural differences. First, the sample distribution is substantially imbalanced (82 effect sizes from Eastern contexts vs. 30 from Western contexts), which may affect the precision and generalizability of the comparison. Second, the “Eastern” category encompasses culturally diverse contexts—including China, Taiwan (China), South Korea, and Iran—that differ markedly in terms of specific cultural dimensions (e.g., collectivism-individualism, power distance, uncertainty avoidance). Third, cultural context was coded at the study level based on the location of data collection; individual participants’ cultural orientations were not directly measured. Thus, while the observed difference is statistically significant, it should be interpreted as a preliminary pattern requiring further investigation with more balanced samples and more refined cultural measures.

Learner Demographics. Learner group type showed a statistically significant moderating effect (F = 8.91, *p* = 0.031). Primary school students exhibited the largest effect size (g = 0.718, 95% CI [0.50, 0.94]), followed by middle school students (g = 0.591), university students (g = 0.542), and non-student or other groups (g = 0.488). H4 is partially supported.

### 4.4. Publication Bias Assessment

**Figure 3:**
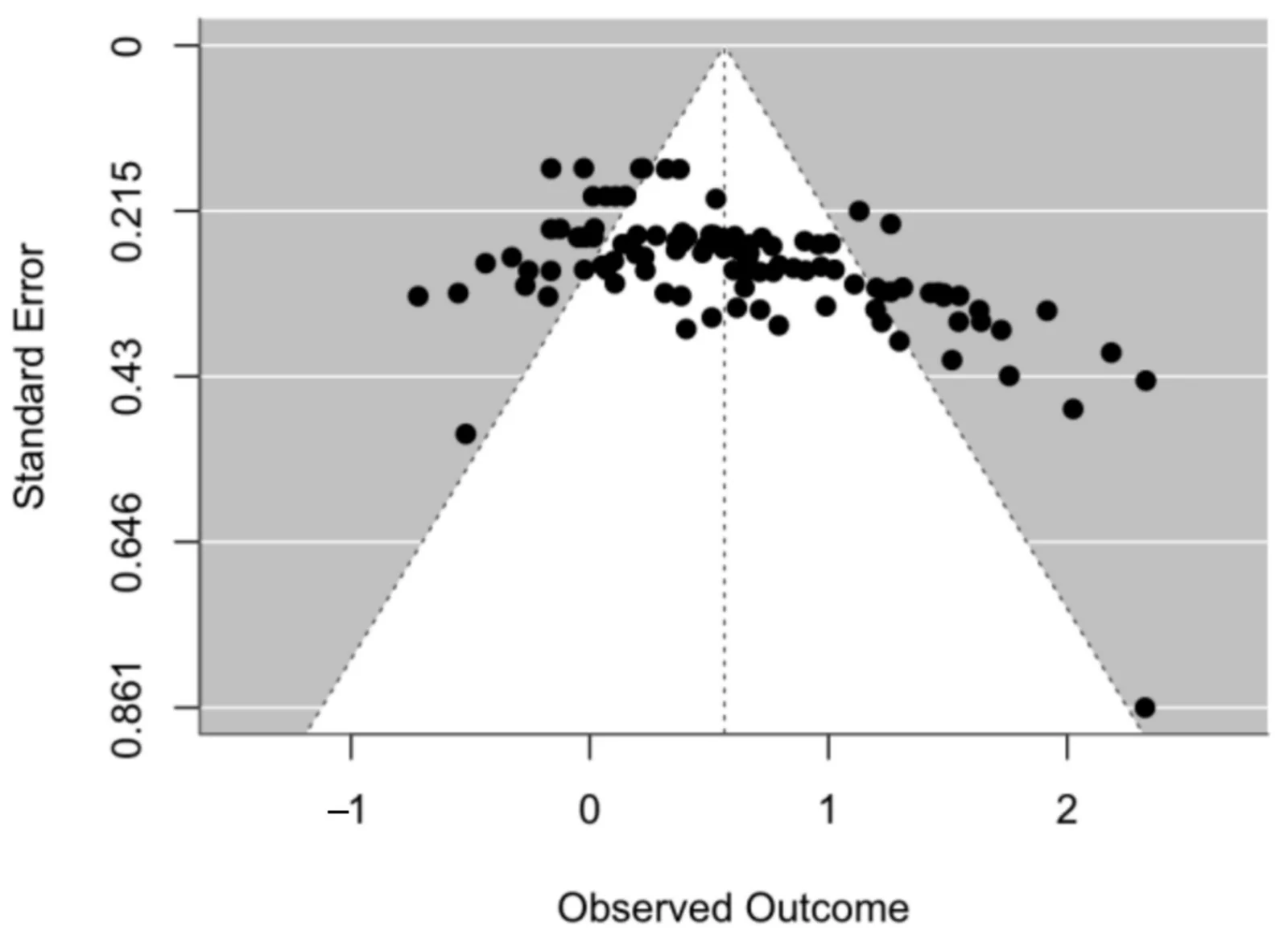
Publication Bias Funnel Plot. Note: Each circle represents an individual study, plotted by its observed outcome (effect size) against its standard error. The grey area indicates the expected 95% confidence interval region around the pooled estimate (or the triangular region within which 95% of studies would fall in the absence of bias) under the assumption of no publication bias.

Figure 3 is a funnel plot that shows an asymmetry in the distribution of the 112 effect sizes; therefore, it may be that there is a considerable publication bias.

Egger’s test confirmed the visual assessment from the funnel plot: intercept β_0_ = 1.87, SE = 0.43, t = 4.35, *p* < 0.001, indicating a systematic tendency for overestimation of effect sizes in studies with lower precision. Publication bias was thus statistically verified. The trim-and-fill method estimated that approximately 9 studies needed to be imputed on the left side of the funnel plot to restore symmetry. The corrected effect size was g = 0.521, 95% CI [0.36, 0.69]. While this corrected estimate is lower than the uncorrected g = 0.607, it remains within the medium effect range, and the lower bound of the 95% confidence interval is substantially above zero. However, we acknowledge the limitations of standard funnel plots and Egger’s test when effect sizes are dependent; these results should be interpreted with appropriate caution. The revised conclusion is: while the presence of significant publication bias suggests that the true effect may be somewhat lower than our unadjusted estimate, the lower bound of the corrected confidence interval remains above zero, indicating that a positive effect persists even after accounting for potential bias.

### 4.5. Sensitivity Analysis

**Figure 4:**
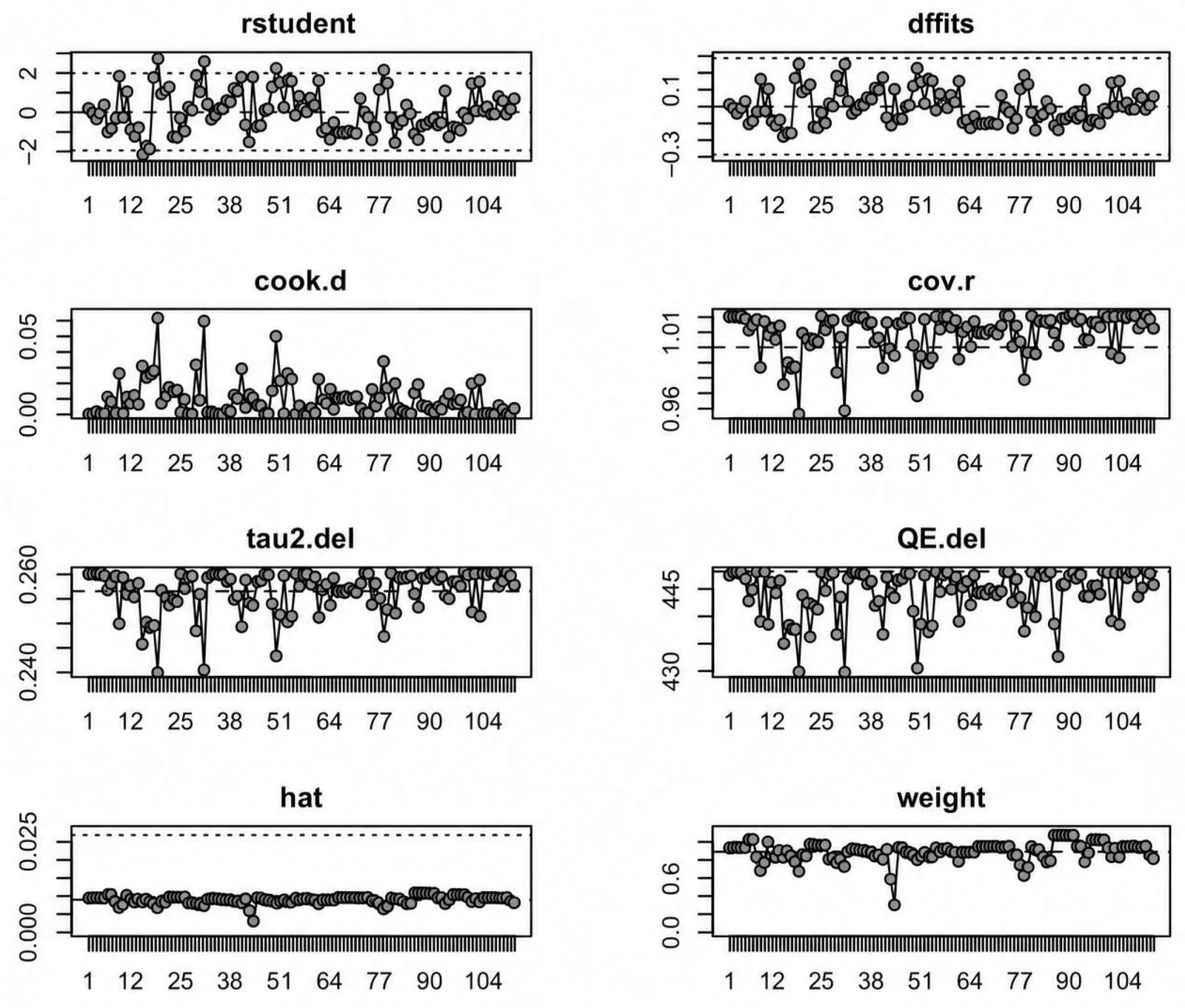
Results of the Influence Diagnosis in the Three-Level Meta-Analysis. Note: Black circles represent each effect size included in the meta-analysis. The dotted lines are horizontal reference lines, these correspond to the reference or critical values associated with the diagnostic indicators.

Figure 4 presents a scatter plot of eight influence diagnostic indicators. All studentized residuals (rstudent) fell within the range of ±3, and the maximum Cook’s distance was approximately 0.06, well below the warning threshold of 1.0. Additionally, DFFITS and leverage (hat) values remained within normal limits, indicating that no individual study had a significant impact on the overall results.

In the leave-one-out analysis, after successively excluding each study, the range of overall effect size estimates was [0.57, 0.64], with neither the effect size nor the statistical significance changing. After excluding Park (2022) specifically (N = 18, vi = 0.741), the effect size was g = 0.598, 95% CI [0.44, 0.75], which closely aligns with the original result. Based on all diagnostic evidence, the conclusions of this study are highly stable and effectively address RQ3.

## 5. Discussion

### 5.1. Theoretical Interpretation of the Main Findings

Based on the results of the three-tier meta-analysis of data from 31 studies, it can be concluded that DPA is positively associated with SLA to a moderate extent (g = 0.607). The three theories—SAT, CLT, and CTML—offer different perspectives on the underlying causes of this relationship; however, they are not contradictory and collectively form a comprehensive explanatory framework.

According to the theory of social agency, DPAs can activate learners’ natural mechanisms for responding to social cues. Through anthropomorphic appearance, synchronous voice, and emotional feedback, learners perceive the agent as a genuine member of society. This sense of social presence is motivating, encouraging individuals to continue learning willingly. The relatively higher agent effect observed among learners from Eastern cultural backgrounds in this study (0.665 vs. 0.473) exemplifies how this phenomenon is amplified in collectivist cultures. In such cultures, people place greater emphasis on social relationships and authority guidance, causing the social presence of agents to evoke stronger psychological responses.

According to the Cognitive Theory of Multimedia Learning, the dual-channel presentation mode of agents leverages the brain’s dual-channel processing system to engage both auditory and visual working memory. Specifically, verbally articulated information is processed through hearing, while visually presented information is processed through sight. This integration of different types of information encoding reduces extraneous cognitive load and frees up cognitive resources for focused language learning. In this study, the highest effect size observed for primary school students was 0.718, indicating that younger students with more limited cognitive resources are more susceptible to the effects of extraneous cognitive load.

According to Cognitive Load Theory, the results of longitudinal studies (0.648) are significantly higher than those of cross-sectional studies (0.421). This difference can be explained by the internal logic of language knowledge accumulation: continuous DPA intervention facilitates the gradual automation of lexical and grammatical schemas through repeated activation, thereby freeing more working memory for constructing higher-order meaning. In contrast, cross-sectional designs cannot induce this cognitive accumulation process after only one exposure. These three theories can be integrated to explain the main effect size observed in this study, forming a coherent and internally consistent framework to understand the influence of various moderating variables.

Based on the current results and comparisons with existing meta-analyses, an effect size of g = 0.607 falls within a reasonable range. For instance, the d = 0.66 reported by Schroeder et al. (2013) for general learning tasks closely aligns with the current estimate, while the g = 0.44 reported by Castro-Alonso et al. (2021) is comparatively lower; both studies were conducted in non-SLA contexts. SLA specifically involves two key components of language learning: declarative knowledge (such as vocabulary and grammar rules) and procedural knowledge (fluent output). Different agents may have varying facilitative effects on the acquisition of these distinct types of knowledge, providing a theoretical foundation for SLA analysis. A three-level variance decomposition indicates that the primary source of total variance is between-study variance (σ^2^_3_), highlighting the need for moderator analysis.

### 5.2. Theoretical Interpretation of Moderating Effects

The moderating effect of the study design type highlights the importance of the time dimension in language learning. The development of language skills is an accumulative process: vocabulary acquisition requires spaced repetition, grammar rules should be reinforced through repeated activation in various contexts, and pronunciation demands frequent practice. A long-term intervention system provides an ideal environment for the continuous process characteristic of longitudinal studies. Based on these findings, the practical implication is that to fully leverage the benefits of DPAs, they should be integrated continuously throughout the teaching process rather than employed in isolated activities.

The significant moderating effect of cultural background warrants careful interpretation. The larger effect observed in Eastern cultural contexts may be tentatively understood through cross-cultural differences in social presence perception. In cultural contexts emphasizing group belonging, authority relations, and interdependent self-construal, DPAs with stable personality characteristics and responsive behaviors may be more readily internalized as genuine social others, generating stronger emotional engagement and cognitive investment. Concurrently, Dang and Liu (2023) found that the relationship between loneliness and robot anthropomorphism was more negative in Chinese participants than in American ones, underscoring the moderating role of cultural context. However, this interpretation remains speculative given the imbalanced sample distribution (82 vs. 30 effect sizes) and the broad categorization of “Eastern” versus “Western” cultures. Hofstede’s cultural dimensions framework offers one possible explanatory pathway, but future research should employ continuous measures of cultural orientation at the individual level rather than relying on country-level binary classifications.

The moderating effect of learner demographics indicates that the age and cognitive development stage of learners influence the effectiveness of intervention measures. Primary school students face two cognitive challenges: the inherent cognitive load resulting from unfamiliarity with the target language and the demands of metacognitive regulation. DPAs can reduce learners’ extraneous cognitive load by providing specific instructions on task execution, timely feedback, and relative ease of use, enabling them to focus more on learning the language material. The relatively limited impact of agent interventions among university students does not imply that agents are ineffective; rather, it may reflect this group’s stronger learning autonomy and cognitive self-regulation abilities, allowing them to develop effective language learning plans independently.

### 5.3. Origins and Responses to Publication Bias

Based on the assessment of publication bias in this study, there is evidence of a systematic positive reporting bias in this field. This phenomenon is relatively common among meta-analyses of educational technology. The reasons include researchers’ focus on substantive effects, journal editors’ preference for publishing “effective” interventions, and the amplification effect of small-sample quasi-experimental designs on effect size estimates. This paper employs transparent disclosure to clearly present the asymmetry pattern of the funnel plot, report the significance of Egger’s test, and use the trim-and-fill corrected effect size as a conservative lower bound for the conclusions. The corrected effect size (g = 0.521) indicates that, even after adjusting for publication bias, the positive effect of DPAs on SLA remains valid and is not based on fundamental flaws. This approach to meta-analysis reporting aims to “honestly indicate the uncertainty without denying that there is a valid signal,” aligning with the open science principles promoted by the current scientific community (Rothstein et al., 2005).

### 5.4. Limitations

Although this paper presents several typical examples, it also has some imperfections.

First, regarding the sample size, the scale of the 31 independent studies is small for certain subgroups (e.g., conference papers, k = 5; non-student learners, k = 8), so these results should be interpreted with caution.

Second, the heterogeneity of outcome measures and agent designs represents a significant limitation. Our analyses treated all SLA outcome measures (vocabulary, grammar, listening, speaking, reading, fluency, accuracy) as a single category, yet these different language skills may respond differently to pedagogical agent interventions. Similarly, agents varied considerably in embodiment, voice quality, emotional expressiveness, interactivity, adaptivity, and feedback type. We were unable to conduct subgroup analyses on these design features because most primary studies did not provide sufficient detail to code these variables reliably. We recommend that future primary studies adopt standardized reporting guidelines for pedagogical agent interventions to enable more fine-grained meta-analytic syntheses. Stratified subset meta-analyses focusing on different language skill types and agent design features are also needed.

Although this paper includes studies up to February 2026, generative AI agents powered by large language models represent a novel type of intervention that has demonstrated some positive outcomes and varying boundary conditions but has not been extensively studied. Currently, there is a relatively limited foundation for independent subset analyses, and next-generation agents in this field should be incorporated into future domain-specific meta-analyses.

## 6. Conclusions

### 6.1. Key Conclusions

Based on a three-level meta-analytic framework and the synthesis of results from 31 independent studies examining the effects of DPAs on SLA and their moderating mechanisms, the primary conclusion is that DPAs have a statistically significant and practically meaningful positive effect on SLA (Hedges’ g = 0.607, 95% CI [0.45, 0.77]). This effect is moderately strong and demonstrates high robustness across three publication bias verification methods and eight sensitivity diagnostic indicators. Furthermore, variations in study design, cultural context, and learner characteristics influence the effect size; specifically, longitudinal designs, students from Eastern cultures, and primary school students tend to exhibit larger effect sizes. These findings align with the theoretical predictions of Social Agency Theory and Cognitive Load Theory.

### 6.2. Practical Implications

For instructional design, the longitudinal moderation effect suggests that DPAs may be more beneficial when integrated as sustained instructional tools rather than as one-off interventions, although this recommendation is based on correlational evidence and should be validated in future controlled trials. With regard to cultural background, our findings indicate a possible trend toward larger effects in Eastern cultural contexts, but given the imbalanced sample and the broad binary classification, we do not recommend making prescriptive design decisions based solely on East–West categorization. Instead, educators and designers should consider local learner characteristics and contextual needs. For young learners (primary school), the larger effect size suggests that DPAs with strong social cues (e.g., anthropomorphic appearance, emotional voice) could be particularly supportive, but this too should be tested with rigorous experimental designs before widespread adoption.

### 6.3. Future Research Directions

The following research directions should be given more weight in the future. First, domain-specific coded meta-analyses should be carried out to study the moderating effects of fine-grained agent design characteristics, such as the degree of anthropomorphic appearance, the type of affective voice and adaptive interaction mode. Second, subset meta-analyses categorized by SLA outcome types (oral fluency, vocabulary breadth, grammatical accuracy, listening comprehension, etc.) would provide more targeted evidence for different language proficiency dimensions. Third, many studies have already been conducted on generative AI agents for a long time, and now a specific meta-analysis of LLM-based agents can be carried out. Fourth, cross-cultural research should employ more refined cultural dimension measures (e.g., continuous analysis of individualism indices) to replace the relatively coarse East–West binary classification. The three-level meta-analytic framework and data structure of this study may serve as a methodological reference for such future investigations.

## Figures and Tables

**Table 1:** Summary of Major Meta-Analyses on Pedagogical Agents.

Author(s) (Year)	N_Studies	k	Effect Metric	Mean Effect	Research Context
Schroeder et al. (2013)	43	43	Cohen’s d	d = 0.66	General learning tasks
Castro-Alonso et al. (2021)	—	—	Hedges’ g	g = 0.44	Multimedia learning (multi-theory framework)
X. Wang et al. (2022)	39	39	g/d	—	Emotion–learning outcome interaction
Dai et al. (2024)	—	—	Hedges’ g	—	Cognitive outcomes in AI simulations
Cheng et al. (2026)	Latest synthesis	Latest synthesis	Hedges’ g	—	Academic performance of generative AI agents
Present study	31	112	Hedges’ g	g = 0.607	Second language acquisition (domain-specific)

Note: “—” means that the study did not report or present a single aggregate mean effect size; k refers to the number of effect sizes; N_studies refers to the number of independent studies.

**Table 2:** Characteristics of Selected Representative Studies in the Meta-analysis.

Author(s) (Year)	Country/Region	Study Design	N	Learner Group	Effect Size (g)
Tai et al. (2020)	China	Longitudinal	49	Middle school	0.71
Carlotto and Jaques (2016)	Brazil	Cross-sectional	39	University	0.48
Zhao et al. (2023)	Taiwan, China	Longitudinal	99	University	0.54
Ahmadi et al. (2017)	Iran	Longitudinal	90	University	2.33 *
Hwang and Zhang (2024)	Taiwan	Longitudinal	56	Middle school	0.63
Rosenthal-von der Pütten et al. (2016)	Germany	Longitudinal	130	Adults	0.39
Chen et al. (2025)	China	Longitudinal	60	Primary school	1.87 *
Choi and Clark (2006)	USA	Longitudinal	74	University	0.44
Lan (2020)	Taiwan	Longitudinal	52	Adults	0.58
Davis and Antonenko (2017)	South Korea	Cross-sectional	107	Primary school	0.36

Note: * indicates large-effect studies (g > 1.5) that were subject to focused sensitivity analysis; g values are Hedges’ g.

**Table 3:** Summary of Moderator Analysis Results.

Moderator	k	Hedges’ g (95% CI)	F	*p*	Level-2 Variance	Level-3 Variance
Study Design			5.83	0.016	0.15	0.31
Cross-sectional (ref.)	18	0.421 [0.21, 0.63]				
Longitudinal	94	0.648 [0.48, 0.82]				
Publication Type			2.14	0.144	0.15	0.31
Journal article (ref.)	87	0.589 [0.43, 0.75]				
Dissertation	18	0.673 [0.38, 0.97]				
Other (conf./report)	7	0.614 [0.21, 1.02]				
Cultural Background			4.27	0.039	0.14	0.30
Eastern (ref.)	82	0.665 [0.49, 0.84]				
Western	30	0.473 [0.27, 0.68]				
Learner Demographic			8.91	0.031	0.08	0.37
University (ref.)	57	0.542 [0.37, 0.71]				
Primary school	30	0.718 [0.50, 0.94]				
Middle school	11	0.591 [0.30, 0.88]				
Non-student/other	14	0.488 [0.22, 0.75]				

Note: k is the number of effect sizes in each subgroup; Hedges’ g and 95% CI are based on the three-level random-effects model (rma.mv); the reference group is the baseline subgroup for each moderator; F is the result of the omnibus test; *p*-values are from two-tailed tests (α = 0.05); Level-2 variance (σ^2^_2_) is the within-study variance among effect sizes; Level-3 variance (σ^2^_3_) is the between-study variance.

## Data Availability

The data supporting the findings of this study are openly available in Zenodo at https://doi.org/10.5281/zenodo.20235639.

## Supplementary Materials

The following supporting information can be downloaded at: https://www.mdpi.com/article/10.3390/bs16091583/s1. References cited in Supplementary Materials include Al Hakim et al. (2022), Chung (2011), Çakmak (2022), Drobisz (2017), Fathi et al. (2024), Flemban (2018), Hong et al. (2014), Ko (2010), Lee (2024), Liu et al. (2023), Lu et al. (2026), Murano (2002), Pan et al. (2025), Steinhaeusser et al. (2022), Tai (2024), C. Wang et al. (2024), and Xiao et al. (2025).
